# Effect of heel elevation on breakover phase in horses with laminitis

**DOI:** 10.1186/s12917-020-02571-5

**Published:** 2020-10-01

**Authors:** Mohamad Al Naem, Lutz-Ferdinand Litzke, Florian Geburek, Klaus Failing, Johanna Hoffmann, Michael Röcken

**Affiliations:** 1 Clinic for Horses (Surgery, Orthopaedics), Faculty of Veterinary Medicine, Justus-Liebig-University, Giessen, Frankfurter str. 108, 35392 Giessen, Germany; 2 Clinic for Horses, University of Veterinary Medicine Hannover, Foundation, Hannover, Bünteweg 9, 30559 Hannover, Germany; 3 Unit for Biomathematics and Data Processing, Justus-Liebig-University, Giessen, Frankfurter Str. 95, 35392 Giessen, Germany

**Keywords:** Heel elevation, Hoof kinetics, Hoof™ system, Horse, Laminitis

## Abstract

**Background:**

In a laminitic horse, the maximal loading of the toe region occurs during the breakover phase. To date, no kinetic data demonstrates the effect of supportive orthopaedic therapy in horses with laminitis on breakover phase. Thus, the purpose of this study was to examine the effect of heel elevation on the breakover phase. Eight horses with acute laminitis treated medically as well as with application of a hoof cast with heel wedge (HCHW) were included in this study. Immediately following cessation of clinical signs of acute laminitis, two measurements using the Hoof™ System were taken: the first with HCHW and the second immediately following removal of the HCHW, i.e. in barefoot condition (BFC). The hoof print was divided into three regions: toe, middle hoof, and heel. Kinetic parameters included vertical force (VF), stance duration, contact area (CA) for all hoof regions during stance phase, duration of breakover, VF in the toe region at onset of breakover and location of centre of force.

**Results:**

The VF and CA were higher in the heel region (63 and 61%, respectively) and decreased significantly after removal of the HCHW (43 and 28% after removal, respectively). The breakover phase in horses with HCHW lasted 2% of stance phase and was significantly shorter than that in BFC, which lasted 6% of stance phase. The VF at onset of breakover for the toe region in horses with HCHW was significantly lower than that in BFC. The centre of the force was located at the heel region in all horses with the HCHW, and at the middle the hoof region in BFC.

**Conclusions:**

Heel elevation in horses with laminitis as examined on a concrete surface significantly shortens breakover phase and decreases the vertical force in the toe region during breakover. HCHW provides adequate support to the palmar hoof structures by increasing the contact area in the heel region and incorporating the palmar part of frog and sole into weight bearing, thus decreasing the stress on the lamellae. Hoof cast with heel elevation could be a beneficial orthopaedic supportive therapy for horses suffering from acute laminitis.

## Background

Acute laminitis is defined as the onset of clinical signs including bounding digital pulse, increased heat at the coronary band in combination with a positive response to the hoof tester over the toe. Moreover, affected horses display a stilted gait and are reluctant to move [1, 2]. The goal of supportive orthopaedic therapy for horses with acute laminitis is to shift the load from the diseased and most painful areas of the hoof to the undamaged areas [3, 4]. Recently, a kinetic study demonstrated that the maximal loading of the toe region in horses with laminitis occurred during breakover phase and that the main shift of the load within the hooves of laminitic horses occurred between the toe and middle hoof regions. Moreover, in laminitic horses, relative vertical force and vertical impulse were higher in the heel region than in other regions, while in the sound horses, relative vertical force and vertical impulse were the highest in the toe region [5, 6].. Thus, easing the breakover phase which is the time from heel-off to toe-off [7], should be considered when treating horses with laminitis to minimize the load on the damaged lamellae in the toe region [6]. A variety of supportive orthopaedics therapy options are available for horses with acute laminitis including hoof cast with heel wedge (HCHW) [3], lily pad, toe bevelling [8], and wooden shoes [9]. However, to the best of our knowledge, no data are available in the literature demonstrating the effects of these treatments on hoof kinetics. Thus, the purpose of this study was to examine the effect of heel elevation on the breakover phase and the vertical force in the toe region during breakover phase as well as the contact area in the heel region in horses with laminitis. We hypothesized that the breakover phase would be shorter in laminitic horses with HCHW as compared to the same horses following removal of the HCHW.

## Results

History and clinical as well as radiographic findings are listed in Tables 1 and 2.

**Table 1:** signalment and anamnestic details of the horses

Number	Breed	Sex	Age (year)	Weight (kg)	Number of laminitic episodes	Reason for laminitis
1	Icelandic horse	Gelding	8	440	1	Colic episode
2	Welsh pony	Mare	13	343	1	NA
3	Warmblood	Mare	15	550	1	Change in exercise routine
4	Icelandic horse	Gelding	10	400	2	NA
5	Welsh pony	Gelding	22	330	2	Cushing’s disease
6	Arabian horse	Gelding	18	410	1	Pasture-associated laminitis
7	Icelandic horse	Mare	15	455	1	Change in exercise
8	Quarter horse	Mare	9	475	1	NA

*NA* Not applicable

**Table 2:** Summary of the results of clinical as well as radiographic examination of the most severely affected forelimb

Number	Obel-grade on admission	Obel-grade after application of HCHW	Degree of phalanx rotation	Sole depth (mm)	Time of hospitalization (days)	Duration of application of HCHW (days)
1	2	1–2	6	11.4	13	12
2	3	2	9	9.3	14	13
3	1	0–1	3	14.3	8	7
4	3	2	7	11	12	11
5	2	1	6	11.4	10	9
6	3	2	10	9.6	18	17
7	2	1	4	11.5	12	11
8	2	1	6	13.6	15	14

There were no significant differences in any of the variables between the left and right forelimbs (t-test for dependent samples). Therefore, the measured variables for both limbs were pooled by using arithmetic mean over both sides.

**Fig. 1:**
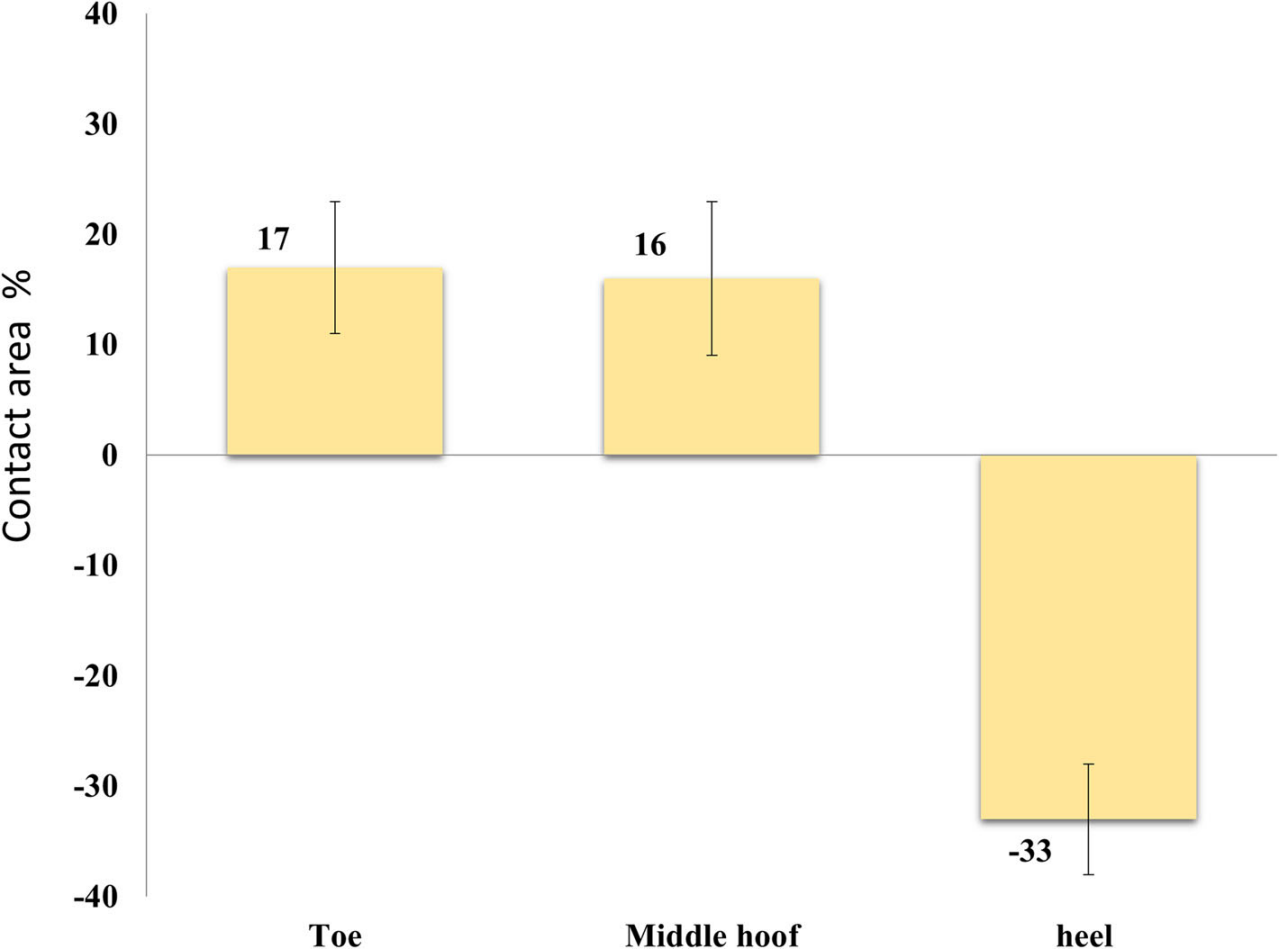
The differences in the percentages of contact area in laminitic horses with and without the HCHW in relation to specific hoof regions

In the toe region, the CA was significantly higher following removal of the HCHW (*p* = 0.002) (Fig. 1). For the VF, there were no significant changes. The VF at tHO for the toe region in horses with HCHW was significantly (*p* < 0.001) lower than those in BFC (Table 3).

**Table 3:** Mean ± standard deviation of the loading and timing variables (VF, CA, VF – tHO, StDur) of eight laminitic horses with and without the HCHW in the toe, middle hoof and heel regions

Variables	Hoof print regions	HCHW	BFC	Difference
**VF (N)**	**Toe**	657 ± 133	756 ± 99	+ 99 ± 65
	**Middle hoof**	290 ± 46	699 ± 87	+ 409 ± 103^a^
	**Heel**	1609 ± 119	1078 ± 113	− 531 ± 43^a^
**VF - tHO**	**Toe**	380 ± 221	682 ± 361 N	− 302 ± 115 ^a^
**CA (cm**^**2**^**)**	**Toe**	13.5 ± 5	16.9 ± 6.9	+ 3.4 ± 5^a^
	**Middle hoof**	6.7 ± 4.3	11.7 ± 4.2	+ 4.4 ± 6^a^
	**Heel**	31.4 ± 7.4	11.1 ± 3.5	−20.3 ± 8^a^
**StDur (s)**	**The entire hoof**	0.76	0.79	0.03

*BFC* Barefoot condition; *HCHW* Hoof cast with heel wedge; *CA* Contact area; *VF* Vertical force; *VF-tHO* Vertical force at onset of breakover, *StDur* Stance duration. ^a^*p* < 0.05

In the middle hoof region, the VF and CA increased significantly following removal of the HCHW (*p* = 0.002, *p* = 0.001, respectively) (Table 3).

**Fig. 2:**
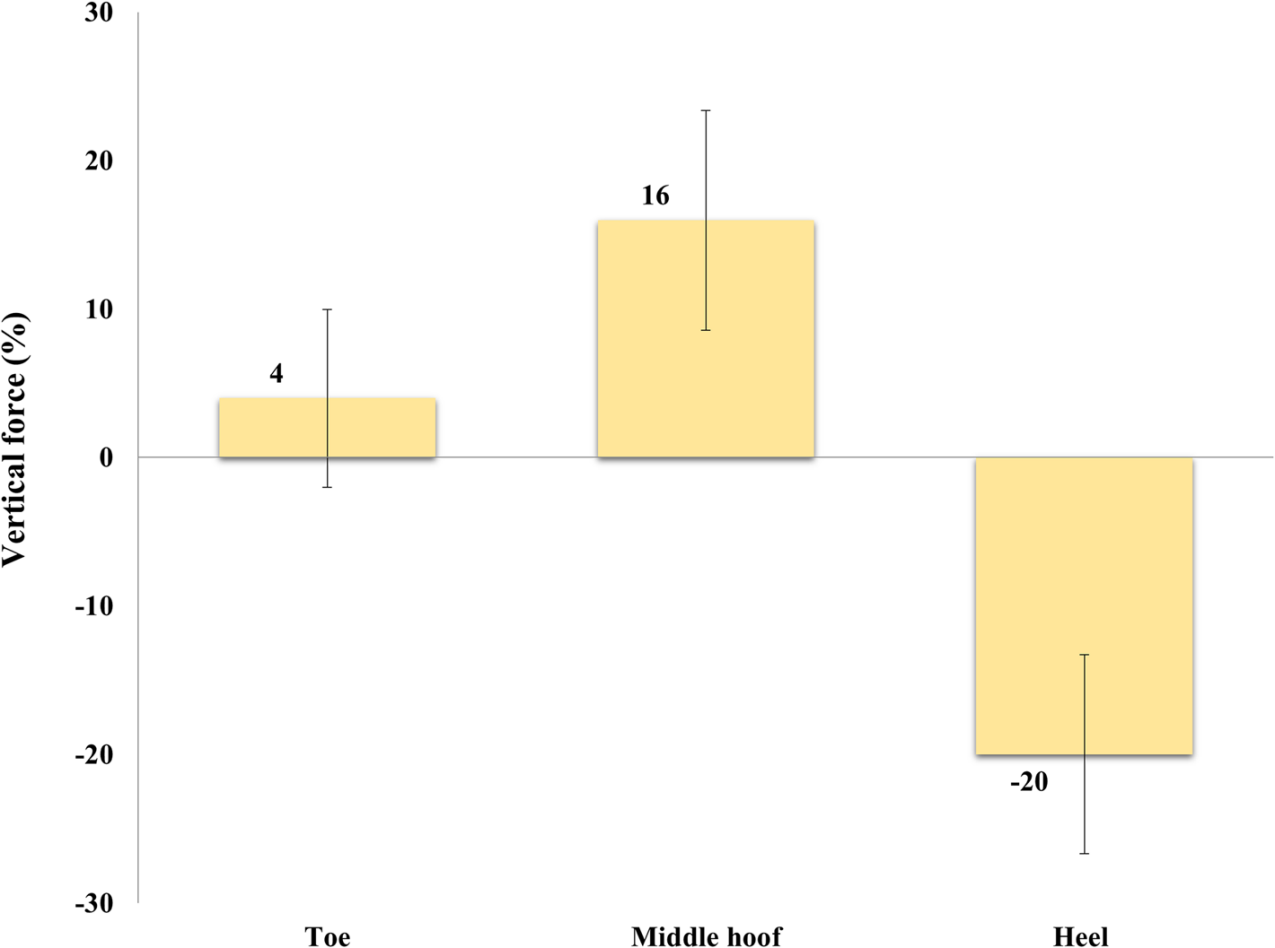
The differences in the percentages of vertical force between laminitic horses with and without the HCHW in relation to specific hoof regions

**Fig. 3:**
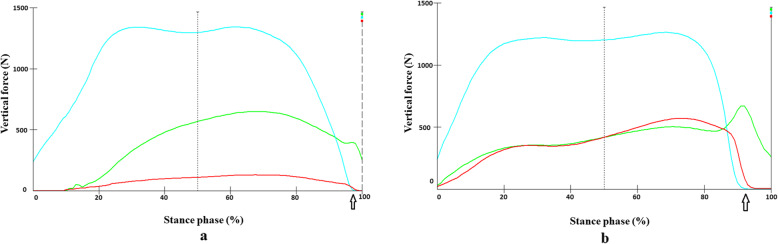
Force-time-curve of the three hoof regions in a representative horse (number 6) with (a) and without an HCHW (b) during stance phase. The heel region (aqua) experienced the highest load in both conditions. The load distributions in the toe (green) and middle (red) regions of the hoof were similar in the BFC. The load in the middle hoof region (red) increased after removal of the HCHW. The arrow indicates the time at which the heel off occurred. BFC = barefoot condition; HCHW = hoof cast with heel elevation

In horses with the HCHW, the VF and CA were higher in the heel region and decreased significantly (*p* = 0.001 and *p* = 0.001 respectively) after removal of the HCHW (Figs. 1, 2). The mean stance phase duration did not significantly differ between the two conditions (HCHW vs BFC) (*p* = 0.38). The breakover phase in horses with HCHW (tHO 98 ± 1.2% of stance phase) lasted 2% of the stance phase and was significantly shorter than those in BFC (tHO 94 ± 1.3%), which lasted 6% of stance phase (Fig. 3).

**Fig. 4:**
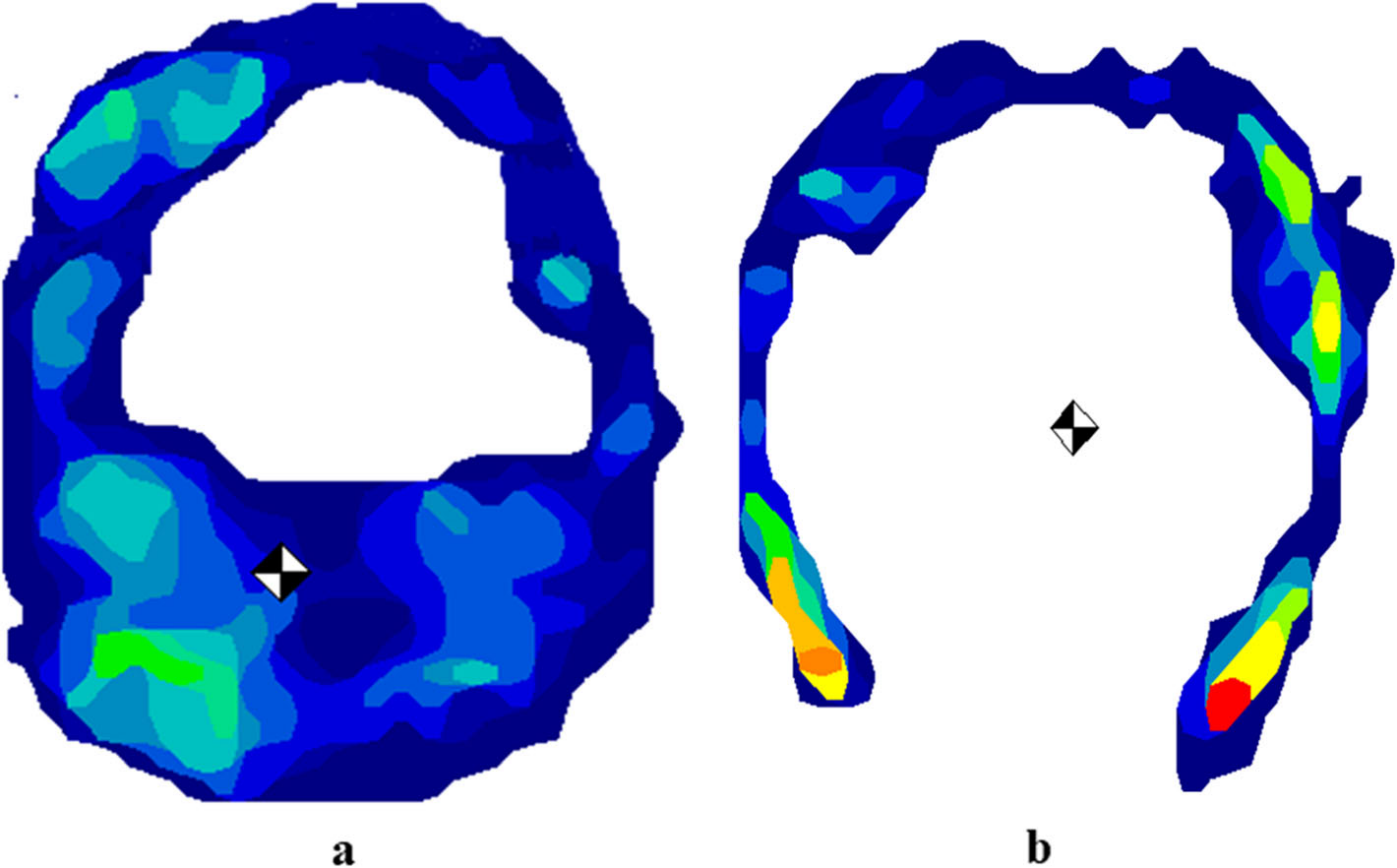
The averaged pressure distribution with the centre of the force **(** **)** of a horse (number 6) with (a) and without the HCHW (b) during the stance phase. Lateral is to the left and dorsal is in the top. The pressure distribution is colour coded: red = highest pressure forces and deep blue = lowest pressure forces, as seen on the pressure scale. With HCHW, the centre of the force was located palmary in the heel region. The hoof contact area decreased after removal of the HCHW in the heel region and the centre of the force shifted dorsally to the middle hoof region compared with HCHW. BFC = barefoot condition; HCHW = hoof cast with heel elevation

The centre of the force was located at the heel region in all horses with the HCHW, whereas it was located at the middle hoof region in laminitic horses in the BFC. A representative example of one forehoof measurement of a horse (number 6) with laminitis following application of the HCHW and in the BFC is shown in Fig. 4.

## Discussion

To the best of our knowledge, the study presented here is the first kinetic study to examine the effect of heel elevation on breakover phase in horses with laminitis.

Our results demonstrate that the breakover phase in laminitic horses with HCHW was significantly shorter than those in BFC. Onset and duration of breakover phase are highly dependent on hoof angle and toe length [7]. Breakover phase is significantly longer in horses with a long toe, as it acts as a long lever arm [10]. Our results can be explained by the fact that heel elevation causes the dorsal hoof wall to be more upright, promoting an earlier breakover. Furthermore, the load of the toe region was significantly lower in horses with HCHW during breakover phase as compared to those in BFC. This is attributed to ease of breakover reducing the time at which the forces acting on the damaged lamellae in the toe region as well as to a palmar shift of the centre of force to the heel region. Consequently, HCHW has the potential to provide relief from pain caused by the damaged lamellae in horses with laminitis.

A recently presented kinetic study demonstrated that in laminitic horses the VF in the heel region was significantly higher as compared to toe and middle hoof regions, whereas the VF in the toe region was highest in sound horses [5]. Therefore, supporting the heel region should be considered when treating laminitic horses. In this context, our results showed that, in horses with an HCHW, the total hoof contact area of the heel region was significantly higher than that observed in the BFC, resulting possibly in more comfort of the horses. Involving the sole and frog by application of an HCHW increased the contact area of the heel region and distributed the load over a larger area. A previous study demonstrated an increase in the weight-bearing contact area, a decrease of total contact pressure, and a decrease of peak contact pressure in healthy horses following the application of a foam sole support [11]. Supporting the sole and frog reduces the weight borne by the wall, thereby reducing the stress on the lamellae [11, 12]. Moreover, all horses in this study were stalled in a box with soft bedding (peat). Placing the laminitic horses on sand or peat is beneficial in supporting the sole thus reducing the load on the hoof wall, as these substrates conform well to the foot [1]. It has been demonstrated that the contact area in clinically sound horses increases to almost double when on deep sand compared to a concrete surface [13]. Therefore, laminitic horses with HCHW would benefit from soft bedding due to increased contact area.

Our results showed that the VF in the heel region of laminitic horses with an HCHW was higher than that observed in the BFC. This finding is in agreement with that of a study demonstrating, that the load on the palmar half of the hoof constitutes 81% of the total load after heel elevation in standing horses suffering from acute laminitis [14]. Furthermore, the current study showed that the centre of force was located in the heel region in horses with HCHW. A palmar position of the centre of pressure and elevation of the heels decrease the moment arm acting on the distal interphalangeal joint, thereby decreasing the stress that the deep digital flexor tendon exerts on the third phalanx [9, 15]. This may help to minimize the stress in the dorsal lamellae and prevent further damage to the damaged lamellae providing ideal conditions for healing.

The proposed theory behind the use of the HCHW is to shift loading forces to the undamaged heel and frog regions to provide relief to the toe region [9]. HCHW should be considered an integral part of supportive therapy for horses with acute laminitis, keeping in mind that the palmar structures of the hoof are capable of bearing more weight [3]. This treatment has been proven to be less suitable in cases with generalized lamellar damage or sinking of the distal phalanx [16]. Moreover, the HCHW prevents hoof wall expansion and deformation which could potentially make the horse more comfortable.

Furthermore, the HCHW is an inexpensive, quick, and easy method to elevate the heel. The plaster bandage can be adapted to any hoof shape and moulded into the frog sulci, increasing the weight-bearing surface area. This distributes the load over a larger area without the need for impression material, which is required in other methods such as wooden shoes and the modified ultimate cuff [4]. However, crushed heels, cracks, or displaced bulbs may develop after long-term application of an HCHW [17]. Elevation of the heels may cause hoof contraction in the long term [18]. In our cases, the application time of the HCHW ranged between 7 and 17 days with no problems reported. As such and depending on our clinical observation, the application of a heel wedge should be of a limited duration (maximal 2–3 weeks).

It has been demonstrated that digital hypothermia would ameliorate experimentally induced laminitis due to the hypometabolic and anti-inflammatory effects [19], although, there is no scientific evidence regarding the effect of digital hypothermia on the treatment of horses with acute laminitis [20]. However, a cooling method entailing a water interface with the hoof that include the distal limb and foot should be used to achieve an effective cooling of the lamellar tissue [21]. The use of such methods would have caused a desintegration of the plaster of paris used in our cases. However, elevation of the heels using other materials could be considered as an option when concurrent cryotherapy is indicated.

This study had some limitations. Ideally, four measurements should have been taken: the first upon admission in the BFC during the acute phase, the second immediately after the application of the hoof cast, and the third and fourth after the acute signs had subsided with and immediately after removal the hoof cast. However, measurement prior to the application of the hoof cast would have taken place during a more acute phase, and would therefore, have been during the more painful and unstable phase of laminitis, as both application and calibration of the measuring system take about 15–20 min. Moreover, horses are required to stand during these procedures on one forelimb with the contralateral limb held off the ground for approximately 1–2 min. Thus, there was a potential risk of further damage to the lamellae, which could have a negative effect on the healing process. Therefore, we only took two measurements, as opposed to four. Other limitations include the small sample size and the variation in breeds included in the study. Laminitis is not a very common disease in horses, so we were forced to include horses from different breeds and sizes. Furthermore, there was not a normal control group in the study; however, the forelimbs from each horse were subjected to two measurements and served as its own control.

## Conclusions

The current study demonstrated that heel elevation in horses with laminitis as examined on a concrete significantly shortens breakover phase and decreases the vertical force in the toe region during breakover. HCHW provides adequate support to the palmar hoof structures by increasing the contact area in the heel region and incorporating the palmar part of frog, sole and into weight bearing, thus decreasing the stress on the lamellae. Hoof cast with heel elevation could be a beneficial orthopaedic supportive therapy for horses suffering from acute laminitis.

## Methods

### Horses

Horses with clinical signs of acute laminitis that were admitted to the Clinic for Horses of the Justus-Liebig-University in Gießen, Germany were included in the study prospectively within a period of 1 year. Inclusion criteria were horses suffering from acute laminitis (initial or recurrence episodes) on both forelimbs with rotation of the distal phalanx. Horses with distal displacement of the distal phalanx, horses affected on only one forelimb or on hindlimbs, as well as those with dorsal capsular rotation were excluded from the study.

**Fig. 5:**
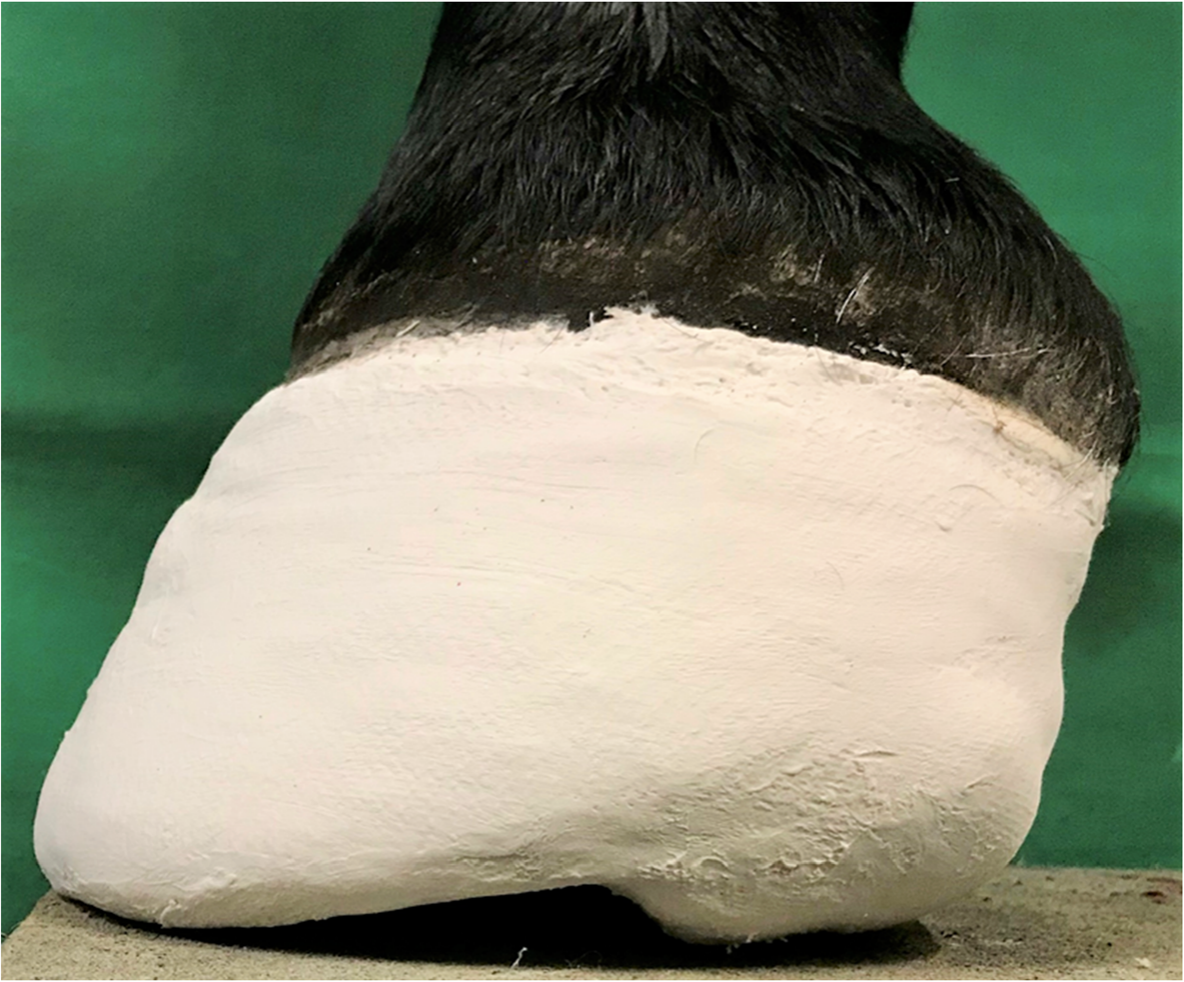
Hoof cast with heel wedge made from Plaster of Paris

Diagnosis of acute laminitis was based on the results of clinical and lameness examination (bounding digital pulse, excessive heat in the feet, positive response to hoof tester and the typical stilted gait) [2], as well as radiographic finding (rotation of phalanx and decreased the sole depth) [22] associated with the acute laminitis. Following diagnosis, lameness was evaluated and graded subjectively according to Obel-grades [23]. If the horses were shod, horseshoes were removed and subsequently, an HCHW was applied on both forelimbs as described previously (supplementary material) [16] (Fig. 5). The gait was re-evaluated subjectively, no kinetic measurements were performed during the acute phase in order to avoid further damage to the sensitive lamellae.

Thereafter horses received medical therapy consisting of meloxicam (0.6 mg/kg, SID orally) acepromazine (0.01 mg/kg, BW, TID orally) and dalteparin-sodium (50 IU/kg, BW, SID, SC) and were stalled in a box with a soft bedding (peat). One horse with equine pituitary pars intermedia disease received pergolide mesylate (2 μg/kg, BW, SID orally). Moreover, all horses received soaked hay 1 kg/100 kg/day. After resolution of the acute signs of laminitis, all medications were discontinued, however the dose of meloxicam was reduced gradually over 2 days and then completely discontinued. The acute episode of laminitis was considered to be overcome when the horses with HCHW were sound at a walk without medication while walking on hard as well as on soft surfaces. Thereafter, the horses were equipped with the Hoof™ System on both forelimbs and two measurements were performed as described in a previous study [6]. The first measurement took place prior to removing the HCHW. The second measurement was performed immediately following removal of the HCHW. Data were acquired at a walk in a straight line on an indoor 3 × 15 m track with a level concrete surface allowing the measurement of 7–11 strides.

### Data processing

**Fig. 6:**
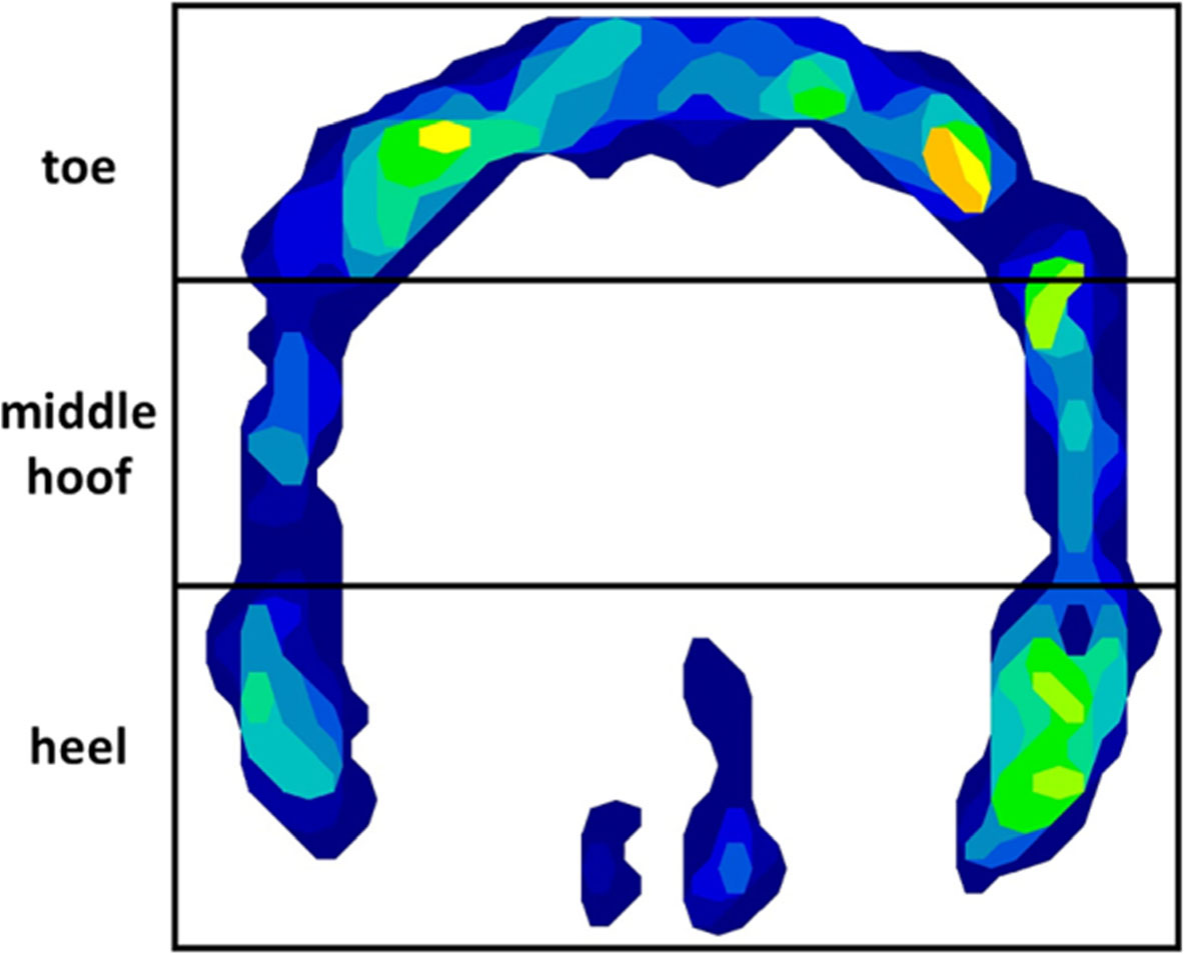
Exemplary hoof print, divided by two lines into the three regions (toe, middle hoof and heel)

To evaluate the kinetic data of the pressure measurement system, the mean of 5 valid strides was calculated and averaged into one pressure image using the FastSCAN Mobile Research software program (version 6.34, TekScan Inc., South Boston). Each individual hoof print was divided into three equal regions by two lines: the toe, middle hoof, and heel (Fig. 6). The following kinetic parameters were calculated and analysed for each region: vertical force (VF) was defined as the force measured by all sensing elements (N and %); hoof contact area (CA) (cm^2^ and %) was defined as the surface area of all loaded sensing elements. Stance phase duration (sec) and the breakover phase, which starts at the time of heel-off (tHO) (% of stance phase), which is the time at which the heel is lifted off the ground, and ends at time of toe-off (end of stance phase), were also recorded. Furthermore, vertical force (Newton) at time of heel-off (VF-tHO) (only for the toe region) was also calculated. Moreover, the location of centre of force was recorded.

### Statistical analysis

For each variable, the data were analysed using two-way repeated-measures analysis of variance (ANOVAs). Side (left and right forelimb) and hoof region were considered as fixed effects. The measured HCHW values were compared to the barefoot values. The difference between the two measurements (the mean value of the HCHW condition minus the mean value of the BFC) for each hoof region was computed and analysed. Afterward, pairwise comparisons were computed for each of the hoof regions using dependent samples *t*-tests, which incorporated the mean square error term of the ANOVA. Type I error inflation was controlled with a Bonferroni-Holm correction. Data were statistically analysed using BMDP2V statistical software (Statistical Software, Los Angeles, CA, USA). *P*-values < 0.05 were considered as statistically significant.

## Supplementary information

**Additional file 1.**

## Data Availability

The datasets used and/or analyzed during the current study are available from the corresponding author on reasonable request.

## Abbreviation

HCHW: Hoof cast with heel wedge
BFC: Bare foot condition
SC: Subcutaneous
VF: Vertical force
tHO: Time of heel-off
VF-tHO: Vertical force at time of heel-off
CA: Contact area
StDur: Stance duration
N: Newton
SID: Once a day
TID: Three time daily
BW: Body weight
sec: Second
