# Supplementary material for: Effect of heel elevation on breakover phase in horses with laminitis
Source: BMC Vet Res. 2020 Oct 1;16:370. doi: 10.1186/s12917-020-02571-5 (PMC7528610; doi:10.1186/s12917-020-02571-5)
Supplement: Supplementary file 1 — Additional file 1. [file 12917_2020_2571_MOESM1_ESM.pdf]

Veterinary Surgery
34:630–636, 2005

# Evaluation Using Hoof Wall Strain Gauges of a Therapeutic Shoe and a Hoof Cast with a Heel Wedge as Potential Supportive Therapy for Horses with Laminitis

NICOLAS HANSEN, MS, HH, FLORIAN BUCHNER, DVM, PhD, JÜRGEN HALLER, Ing., and GERHARD WINDISCHBAUER, DI Dr

**Objective**—To evaluate using strain gauges, a hoof cast with heel wedge, and a therapeutic shoe with unsupported toe for their effectiveness in redistribution of load from the dorsal hoof wall.
**Study Design**—In vitro biomechanical study.
**Sample Population**—Twenty forelimb specimens.
**Methods**—Rosette strain gauges were placed on the dorsal and lateral hoof wall of 20 normal shaped hooves. Limbs were loaded vertically using a tensile testing machine with a 1 Hz sinusoidally cycling load up to 3000 N during 15 seconds. Mean values of principal strain and direction at 2500 N load were calculated for 3 experimental conditions (unshod, therapeutic shoe with unsupported toe, and hoof cast with heel elevation) and tested by ANOVA ($P < .05$).
**Results**—Vertical limb loading in an unshod hoof leads to a biaxial compression of the dorsal wall with high longitudinal compression ($\varepsilon_2 = -1515\,\mu m/m$). Principal strain at the dorsal wall ($\varepsilon_2$) was decreased by 23% with the therapeutic shoe and by 59% with the hoof cast. On the lateral hoof wall principal strain was unchanged with the shoe, but increased by 34% with the cast.
**Conclusions**—Strain measurements indicate unloading of the dorsal hoof wall by both methods with the cast being more effective than the shoe.
**Clinical Relevance**—The hoof cast with wedge offers substantial unloading of the dorsal wall, but increases load on the quarter. Therefore a hoof cast would likely be most helpful in acute laminitis when palmar structures can still bear load. The therapeutic shoe offers rehabilitation and regrowth of the dorsal wall without increased load on the quarter wall.
© *Copyright 2005 by The American College of Veterinary Surgeons*

**Key words:** horse, laminitis, strain gauges, hoof wall, laminitis therapy, therapeutic shoe, hoof cast.

## INTRODUCTION

EQUINE LAMINITIS is a complex systemic disease that involves digital vascular changes, ischemia, and necrosis of the dermal lamellae.[1] Lamellar damage results in a breakdown of the interdigitating system of primary and secondary lamellae with subsequent separation of the distal phalanx from the hoof wall.[2] Depending on the extent of damage and the biomechanical forces acting on the hoof, the distal phalanx can "sink" (vertical displacement) or "rotate", or the hoof capsule can slough completely. Acute laminitis is an emergency that requires systemic treatment and specific management of the feet.[3,4] Therapy is based on intensive medical care that tries to eliminate or minimize any causative factor, provide analgesia, and limit damage at the lamellar junction.[2,4–6]

From the Department for Small Animals and Horses and the Department for Natural Sciences, University of Veterinary Medicine Vienna, Vienna, Austria.
Supported by a grant of the Hochschuljubilaeumsstiftung H-83/99, Vienna, Austria.
Address reprint requests to Dr. HHF Buchner DVM, PhD, Department for Small Animals and Horses, University of Veterinary Medicine Vienna, Veterinaerplatz 1, A-1210 Vienna, Austria. E-mail: Florian.Buchner@vu-wien.ac.at.
Submitted October 2004; Accepted January 2005
© Copyright 2005 by The American College of Veterinary Surgeons
0161-3499/04
doi:10.1111/j.1532-950X.2005.00023.x

Hoof support that reduces stress on the dorsal hoof wall is considered important to prevent or minimize progression of lamellar injuries, prevent compression of the circumflex artery and solar plexus, and reduce pain.[7] In addition to shoe removal and stall confinement on deep bedding, there is general agreement that heel elevation to decrease the pull of the deep digital flexor tendon (DDFT) and support of the palmar areas of hoof and frog to decrease stress on the hoof wall are important.[3,7–10] Reduced stress on the dorsal wall can be accomplished by recruitment of all other parts of the ground surface of the hoof, using soft bedding or pads made from soft material like gauze or styrofoam, or special support of the frog using commercial pads. Furthermore, dorsal wall stress can be reduced by toe beveling, which decreases the leverage of the toe at break over and the stress on the coronary band.[7,11]

Heel elevation is used to decrease stress imposed on the lamellar tissues by flexing the distal interphalangeal joint (DIP) and shortening the moment arm acting on the DIP.[12] This decreases the flexion moment provided by the DDFT reducing the pull of the DDFT and decreasing tensile stress in the dorsal lamellae.[7,9,13] A hoof cast described by Huskamp[14] combined heel elevation with support of the frog, bar, and crus of the sole. The hoof cast has a palmar wedge (Fig 1A) that shifts the point of the force applied to the quarter and frog slightly palmar of the DIP rotation center with the intent of relieving the dorsal wall and reducing tensile forces from the DDFT. However, the actual forces resulting from application of the hoof cast are unknown.

Displacement of the pedal bone leads to compression of the coronary corium, which results in reduced perfusion of the corium and decreased growth of the dorsal hoof wall.[11,15,16] Restoration of normal hoof shape is a therapeutic goal achieved by trimming, and support devices like frog pads and orthopedic shoes for realignment of the distal phalanx.[4,9] To encourage normal hoof growth, unloading of the dorsal aspect of the hoof wall is recommended to release stress on the coronary corium. Dorsal hoof wall resection ensures maximal release of the coronary corium, whereas grooving the dorsal hoof wall is a less radical method that promoted dorsal hoof wall growth in chronic laminitis, when performed in conjunction with corrective trimming and shoeing.[17,18] Another approach reported by Moyer[19] leaves the toe unsupported by a combination of trimming and shoeing where the shoe is positioned well caudally on the heel and a space is created between toe region and shoe to relieve the dorsal aspect of the wall. However, the degree of strain reduction at the dorsal wall has not been quantified.

Stress and strain characteristics of the hoof wall during loading, as well as effects of support devices can be measured using the strain gauge technique.[20,21] Repeatable patterns of strain on the hoof wall can be measured in vivo or in vitro.[20–23] Thus, our objective was to determine in vitro, by application of strain gauges, hoof wall deformation and the effectiveness of a therapeutic horse shoe with unsupported toe and supported quarter (adapted from Moyer[19]) and a hoof cast with palmar wedge (Huskamp[14]). The hypothesis tested was that both hoof cast and therapeutic shoe will reduce dorsal wall compression and increase lateral wall compression.

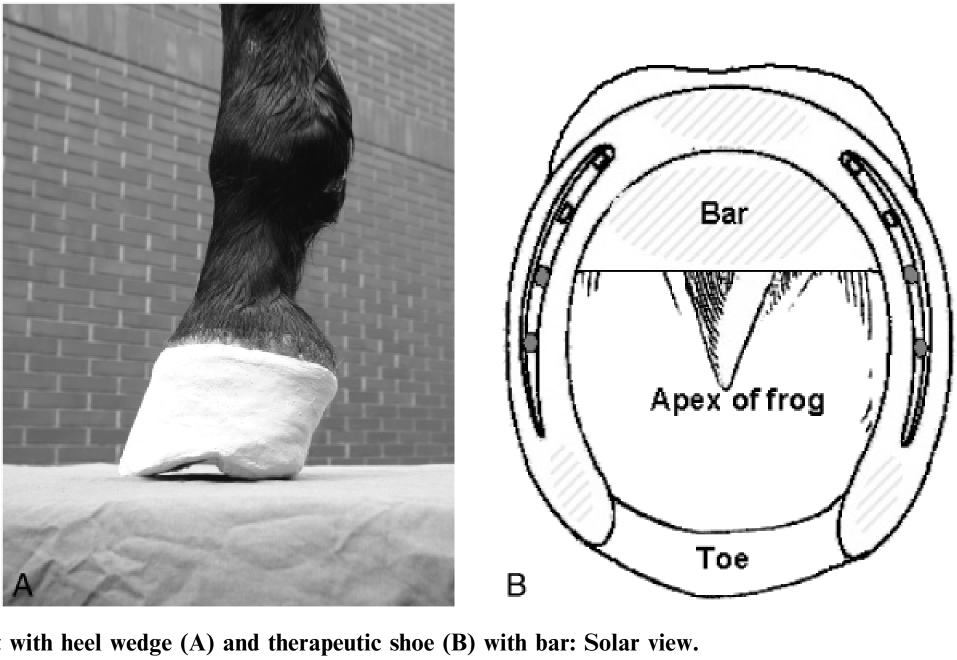


**Fig 1. Hoof cast with heel wedge (A) and therapeutic shoe (B) with bar: Solar view.**

## MATERIALS AND METHODS

The distal aspect of the forelimbs (transected at the antebrachiocarpal joint and preserving the check ligament of the DDFT) from 20 horses that died for reasons unrelated to the locomotor system were collected within 12 hours and stored in plastic bags at −18°C. Limbs were thawed at room temperature 18 hours before study and the hooves were professionally trimmed.

### *Strain Gauge Application*

Encapsulated-rosette-type strain gauges with foil structure and 3 measuring grids (type 1-RV91-3/120, Hottinger Baldwin Messtechnik (HBM), Darmstadt, Germany) were used to measure the local strain in 3 directions (0°, 45°, 90°) for calculation of principal strain. The dorsal hoof wall gauge ($G_1$) was positioned on the midline of the dorsal wall at 40% of the distance between the coronet and ground surface (Fig 2A). The lateral hoof wall gauge ($G_2$) was attached at the lateral quarter of the hoof at 20% of the distance from the lateral angle to the medial angle of the wall, and at 40% of the distance between the coronet and ground surface. Before bonding the strain gauges, the hoof wall was sanded with fine grade sand paper and degreased with acetone and isopropyl alcohol. Strain gauges were attached to the wall with a 2 component adhesive on a base of methylmethacrylate (X60 Rapid Adhesive, HBM). The primary grid (A) of the gauge was aligned parallel to the hoof tubules (0°), so strain direction could be determined with respect to the hoof tubules. The 2 other grids of the gauge were orientated at 45° (grid B) and 90° (grid C) to grid A. Gauges were covered with protection foil with kneading compound (ABM 75, HBM) to prevent any external mechanical influence. Because the gauges are temperature sensitive, room temperature and surface temperature of the hoof were recorded by 2 sensors during experiments.

### *Materials Testing*

Each forelimb was mounted on a tensile testing machine. The limb was attached by a bolt through the proximal end of the third metacarpal bone. Each limb was loaded in vertical direction with a 1 Hz sinusoidally cycling load with amplitudes up to 3000 N during 15 seconds, similar to maximal forces exerted during normal walk.[24] Limbs were subjected to an increase in load to 2000 N, followed by 10 cycles with loads alternating between 1200 and 3000 N, after which the load was reduced to end the trial. This loading protocol to test hoof wall deformation was performed 4 times for each hoof in the following sequence: (1) therapeutic horse shoe with unsupported toe (Moyer); (2) 1st control; (3) hoof cast with palmar wedge (Huskamp); and (4)2nd control.

### *Hoof Support Devices*

The therapeutic shoe was a reversed shoe with a bar to shift support to the quarter and palmar aspects of the frog. The bar ended approximately 3 cm palmar to the apex of the frog. The space between the bar and frog was filled with compressed tow for even distribution of the support. The shoe ended at the border between dorsal and lateral hoof wall, and led to an unsupported toe (Fig 1B) consistent with Moyer.[19]

The hoof cast we examined was made from 2 rolls of plaster cast (Biplatrix, BSN medical SAS, Vibraye, France).[14] The first third of 1 roll of cast material was unrolled and carefully molded into the collateral and central grooves of the frog until the support was some millimeters higher than the weight bearing surface of the hoof wall. The rest of the plaster cast was placed as a roll from quarter wall to quarter wall leaving 2 cm of the apex of the frog unsupported, resulting in a wedge between hoof and ground with an angle between 15° and 20°. The 2nd roll of cast material was used to secure the wedge by encasing the hoof and the wedge in cast material (Fig 1A).

The control condition, an unshod hoof without any appliances, was tested twice, once after the shoe was applied and

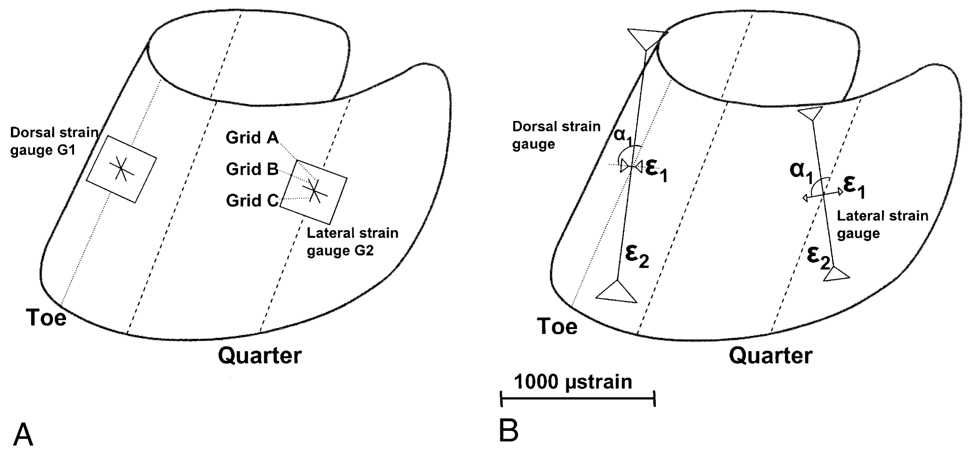


**Fig 2. Stylized representation of the hoof wall. (A) Localization of dorsal and lateral strain gauge at the toe and quarter wall, as well as orientation of the grids (A, B, C) relative to the horn tubules. (B) Principal strains $\varepsilon_1$ and $\varepsilon_2$ and orientation $\alpha_1$ at the dorsal and lateral wall of a bare hoof with 2500 N vertical loading.**

again after the wedge cast, to determine if degradation of the specimen had occurred because of time.

### *Data Acquisition and Analysis*

For data acquisition, an 8 channel carrier-frequency amplifier (Spider 8, HBM) with a 16 bit A/D converter was connected to a computer. Sampling rate was 600 Hz. Before each measurement, the amplifiers were set to 0 without any load applied to the hoof. Principal strain amplitudes ($\varepsilon_1$ and $\varepsilon_2$) and directions ($\alpha_1$) were calculated from the single grid data by software (Catman, HBM). The orientation of $\varepsilon_1$ is defined by the orientation angle $\alpha_1$ (angle between $\varepsilon_1$ and tubules); $\varepsilon_2$ is orientated perpendicular to $\varepsilon_1$ (Fig 2B). All recordings were checked for irregular results of single gauges. Recordings showing signal errors (i.e., exceeding the maximal limits of the gauges) were discarded.

### *Statistical Analysis*

Data acquired at 2500 N were averaged for each recording. Principal strain amplitudes and their directions for all hooves were summed using descriptive statistics. Amplitudes and directions of the 2 control conditions were compared using a paired samples *t*-test. The control condition and the 2 treatment conditions were tested using repeated measures ANOVA (SPSS 11.5 Lead Technologies, Chicago, IL). If a significant influence was found a paired samples *t*-test was performed to compare the individual treatments with each other. Significance level was set at $P < .05$.

## RESULTS

Hoof temperature varied between 16°C and 18°C, which was assumed to be sufficiently constant. Strain signals from the dorsal hoof wall gauges were mostly linear (Fig 3) whereas signals from some gauges, especially at the lateral hoof wall, had non-linear behavior (i.e., with increasing load, initial compression was followed by extension or vice versa). The calculated principal strains, representing the signals of the 3 grids of a rosette, had linear characteristics, being either compressive ($\varepsilon_2$, $\varepsilon_1$) or tensile ($\varepsilon_1$; Fig 3). For the 2 control recordings, there were no significant differences for $\varepsilon_2$, for the angle of the principal strains, and for $\varepsilon_1$ at the lateral wall. Only $\varepsilon_1$ at the dorsal wall had a small, but significant increase in strain from −51 to 36 μm/m.

### *Principal Strain Magnitudes and Direction of the Dorsal Hoof Wall Strain Gauges*

The calculated principal strain was a biaxial compression of the dorsal hoof wall. For the unshod hoof, a strong longitudinal compression ($\varepsilon_2 = -1515$ μm/m) and a small compression perpendicular to $\varepsilon_2$ ($\varepsilon_1 = -51$ μm/m) was observed at 2500 N (Table 1). The principal strain ($\varepsilon_2$)

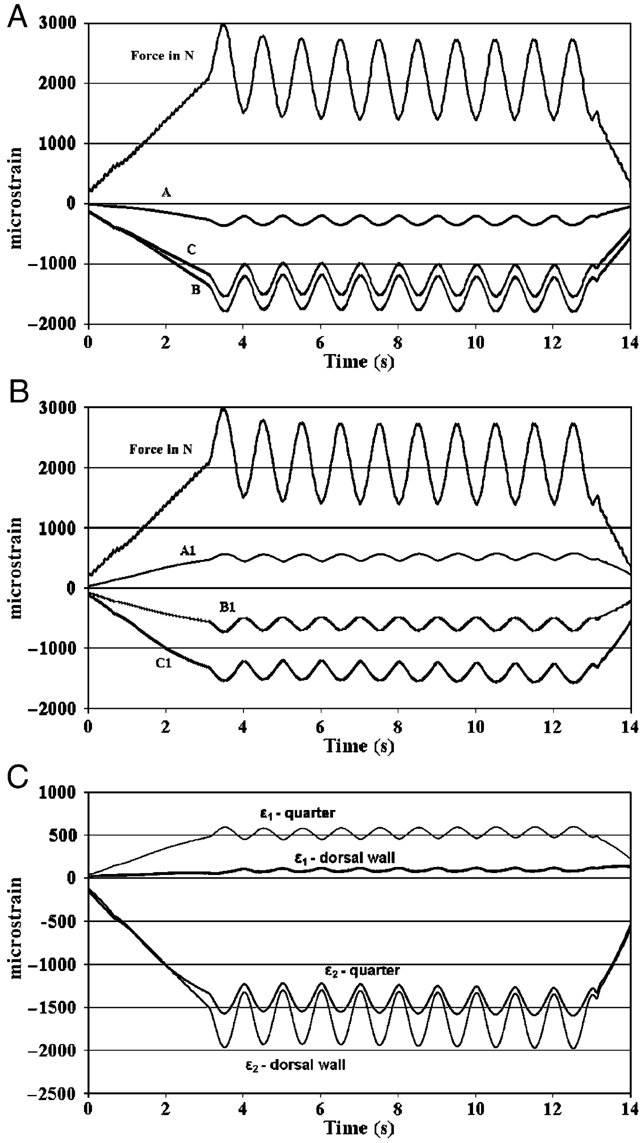


**Fig 3. Vertical force and strain patterns of the single grids. (A) at the dorsal wall (A, B, C), (B) at the quarter (A1, B1, C1), and (C) principal strain $\varepsilon_1$ and $\varepsilon_2$ at dorsal wall and quarter in an unshod hoof during in vitro loading.**

on the dorsal wall was decreased by 23% with the Moyer shoe and by 59% with the hoof cast. The principal strain ($\varepsilon_1$) changed from slight compression (unshod hoof) to tension (cast, laminitis shoe); however, the direction of the principal strain did not change significantly.

### *Principal Strain Amplitudes and Direction of the Lateral Hoof Wall Strain Gauges*

The calculated principal strain of the unshod hoof had a large longitudinal compression ($\varepsilon_2$) and a slight tension

Table 1. Mean ( ± SD), Principal Strains ($\varepsilon_1$, $\varepsilon_2$), and Strain Orientation ($\alpha_1$) of 20 Hooves at 2500 N Vertical Load Unshod (Control 1 and 2) and with Therapeutic Shoe and Hoof Cast

| | Therapeutic shoe | Control 1 | Hoof cast | Control 2 |
|---|---|---|---|---|
| Dorsal wall | | | | |
| $\varepsilon_1$ (μm/m) | $235^b$ (553) | $-51^a$ (568) | $360^b$ (292) | $36^*$ (533) |
| $\varepsilon_2$ (μm/m) | $-1161^b$ (506) | $-1515^a$ (534) | $-625^c$ (323) | −1612 (636) |
| $\alpha_1$ (degree) | 104 (79) | 108 (70) | 80 (46) | 108 (70) |
| Quarter wall | | | | |
| $\varepsilon_1$ (μm/m) | 108 (269) | 204 (288) | 176 (528) | 231 (264) |
| $\varepsilon_2$ (μm/m) | $-875^a$ (589) | $-966^{ab}$ (875) | $-1297^b$ (586) | −1029 (787) |
| $\alpha_1$(degree) | 91 (47) | 119 (53) | 85 (44) | 112 (63) |

Different superscripts denote significant different values ($P < .05$) between different conditions.
*Denotes significant differences between both control conditions.

in $\varepsilon_1$ at the lateral hoof wall. There were no significant differences in $\varepsilon_1$ between the test conditions. For $\varepsilon_2$, a significant difference was observed between the cast and unshod hoof. With the hoof cast, $\varepsilon_2$ increased by 33% compared with the unshod hoof, whereas there was no significant difference between the unshod hoof and the Moyer shoe. The orientation of the principal strains was not significantly different.

## DISCUSSION

Loading of the equine limb causes characteristic changes in hoof shape, as well as a deformation of the hoof wall, which can be measured by strain gauges.[20,21] Strain measurements for the unshod normal hoof had biaxial compression of the dorsal hoof wall, with a principal strain orientation nearly parallel to the horn tubules. These results agree with the in vivo results of Thomason et al and reflect the combined effect of the vertical load and compression perpendicular to the tubules because of a palmar/plantar movement of the proximal dorsal wall.[23,25] The same agreement between our results and in vivo measurements was observed in the quarter wall, where the principal strain $\varepsilon_2$ was also compressive, but with slight tension in $\varepsilon_1$. This finding can be explained by vertical compression of the wall without other major compressive forces because of shape changes.

The validity of results from in vitro studies may be limited by degradation of the hoof wall material, the lamellae, and limb structures. We used 2 methods to control for potential degradative effects: (1) comparisons between reported in vivo data and our in vitro results and (2) repeated control measurements on unshod hooves during the experiment. Comparison of our results with Thomason et al[23] indicate good agreement both in quality and in strain magnitudes, indicating that the in vitro data can be used as a model for the in vivo situation. Further, results from the 2 control tests had high repeatability with no significant differences except from $\varepsilon_1$ of the dorsal wall, where there was a very small but significant increase in tension. However, compared with the differences between the 2 treatment conditions and the unshod hooves this increase was considered to be of minor influence.

Unlike the loading pattern experienced during a complete stance because of changing loading direction, our study design only tested vertical loading (i.e., similar to midstance loading during in vivo measurement). Impact loading and spikes at breakover cannot be simulated using this approach; however, we assumed that load changes during midstance were sufficiently representative of the general loading pattern of the hoof.

The Huskamp[14] cast for hoof support during acute laminitis caused a substantial decrease in compression of the dorsal hoof wall. Two factors were responsible for this strain reduction: (1) a vertical load reduction in the dorsal wall and load transfer to palmar structures and (2) a decrease in the pull of the DDFT. The vertical load exerted by body mass acts on the distal phalanx causing biaxial compression of the dorsal wall with maximal compression in a direction nearly parallel to the horn tubules. The connection between the wall and the distal phalanx is the interface between epidermal and dermal lamellae, and collagen fibers between lamellae and the bone surface. A decrease in nearly 60% of the longitudinal compression of the dorsal wall indicates therefore a substantial decrease in forces within the lamellar connection and correspondingly less risk for disruption of epidermal and dermal lamellae also. However, this relief of the dorsal wall is based on an increase in the load on the palmar structures with a 30% increase in compression in the quarter wall. If the palmar structures of the hoof: quarters, frog, bar, and sulci are less damaged in a horse with acute laminitis and capable of bearing more weight, then the use of the hoof cast could help minimize damage to the dorsal wall and likely decrease pain. This would be suitable for less severe cases of laminitis, where no generalized lamellar damage and sinking of the distal phalanx has occurred. For sink-

ers, palmar load transfer is not an option and more fundamental limb support, such as limb casts or support by a sling is necessary.[14]

A second effect of the Huskamp cast, in addition to load transfer to the palmar hoof structures, is a decrease in the pull of the DDFT caused by heel elevation. Forces of the DDFT are dependent on the distal interphalangeal (coffin) joint angle and can be significantly reduced by heel wedges.[10,13,26] Similar to the hoof cast, shoes with 10–18° wedges[9] or clogs with wedges (Dallmer, Salzhausen-Putensen, Germany) are used to achieve this reduction in pull of the DDFT. So, does elevation of the heel cause increased and harmful shear stress in the dorsal lamellae? An increase in dorsal wall angle brings the direction of the horn tubules closer to vertical loading. Shear forces are the main component of the forces between dermal and epidermal lamellae and therefore the main component of dorsal wall strain. Loading of the hoof with a cast with a heel elevation similar to the 18° wedges used by Redden,[9] however, did not increase the principal strain at the dorsal wall, but decreased it by nearly 60%. Therefore, elevation of the heel with support of the palmar structures seemingly would not increase the risk of lamellar damage because of shear stress, but should unload the lamellar junction.

Similar to the hoof cast, the therapeutic shoe with an unsupported toe caused reduced compression of the dorsal wall, but to a much lesser extent. Only 23% of the principal strain $\varepsilon_2$ was reduced; however, there was no increase in principal strain in the quarter wall. Obviously, there was a load transfer from the dorsal wall to the other palmar structures of the hoof, such as quarter, heel, bar, and frog, without increasing the load on the quarter wall. Two factors may contribute to these observations: (1) the hoof cast concentrated the load to a smaller area at, and between, the quarter walls, whereas the therapeutic shoe supported the whole quarter and heel region and (2) there was no change in the angle of the hoof to the ground, as there was no wedge in the therapeutic shoe. Therefore, the therapeutic shoe had less influence on hoof mechanics than the cast, but could potentially be an advantageous long-term solution to promote dorsal horn growth without overloading other hoof areas. Similar relief of the coronet of the dorsal wall is thought to occur by coronary grooving and more radically by dorsal wall resection.[17,18,27] The degree of distal phalanx rotation and amount of damaged tissue may be important determinants of which method of dorsal unloading would be the most beneficial therapeutic manipulation for an individual horse.

Both treatment approaches unloaded the dorsal wall and the hoof cast more effectively than the therapeutic shoe. Whereas the hoof cast should offer good support in acute laminitis without severe digital collapse, the therapeutic shoe should promote long-term rehabilitation and regrowth of the dorsal wall horn.

## REFERENCES


1. Hunt RJ: A retrospective evaluation of laminitis in horses. Equine Vet J 25:61–64, 1993
2. Pollitt CC, Kyaw-Tanner M, French KR, et al: Equine Laminitis. Proc Am Assoc Equine Practnr 49:103–115, 2003
3. Redden RF: Shoeing the laminitic horse. Proc Am Assoc Equine Practnr 43:356–359, 1997
4. Yelle M: Clinicians guide to equine laminitis. Equine Vet J 18:156–158, 1986
5. Brumbaugh GW, Lopez HS, Hoyos Sepulveda ML: The pharmacologic basis for the treatment of developmental and acute laminitis. Vet Clin North Am Equine Pract 15:345–363, 1999
6. Chapman B, Platt GW: Laminitis. Proc Am Assoc Equine Practnr 30:99–115, 1984
7. Parks AH, Balch OK, Collier MA: Treatment of acute laminitis: supportive therapy. Vet Clin North Am Equine Pract 15:363–375, 1999
8. O'Grady S: A practical approach to treating laminitis. Vet Med 88:867–875, 1993
9. Redden RF: 18° Elevation of the heel as an aid to treating acute and chronic laminitis in the equine. Proc Am Assoc Equine Practnr 38:375–379, 1992
10. Willemen MA, Savelberg HHCM, Barneveld A: The effect of orthopaedic shoeing on the force exerted by the deep digital flexor tendon on the navicular bone in horses. Equine Vet J 31:25–30, 1999
11. Goetz TE: Anatomic, hoof and shoeing considerations for the treatment of laminitis in horses. J Am Vet Med Assoc 190:1323–1332, 1987
12. Eliashar E, McGuigan MP, Wilson AM: Relationship of foot conformation and force applied to the navicular bone of sound horses at the trot. Equine Vet J 36:431–435, 2004
13. Thompson KN, Cheung TK, Silverman M: The effect of toe angle on tendon, ligament and hoof wall strains in vitro. J Equine Vet Sci 13:651–654, 1993
14. Huskamp B: Anmerkungen zur orthopädischen Behandlung der Hufrehe. Pferdeheilkunde 6:3–9, 1990
15. Eustace RA, Caldwell MN: Treatment of solar prolapse using the heart bar shoe and dorsal hoof wall resection technique. Equine Vet J 21:370–372, 1989
16. Herthel D, Hood DM: Clinical presentation, diagnosis, and prognosis of chronic laminitis. Vet Clin North Am Equine Pract 15:375–395, 1999
17. Bolz W: Behandlung der chronischen Hufrehe. Tierärztl Rundsch 45:651–656, 1939
18. Ritmeester AM, Ferguson DW: Coronary grooving promotes dorsal hoof wall growth in horses with chronic laminitis. Proc Am Assoc Equine Practnr 42:212–214, 1996
19. Moyer W: Corrective shoeing. Vet Clin North Am Equine Pract 2:3–24, 1980
20. Knezevic PF: Klinik des Trachtenzwanghufes und Grundlagen der Ungulographie mit Dehnungsmessstreifen beim

Pferd. Wien Tierärztl Monatsschr 49:777–824, 869–904, 944–959, 1962
21. Colles CM: A technique for assessing hoof function in the horse. Equine Vet J 21:17–22, 1989
22. Preuschoft H: The external forces and internal stresses in the feet of dressage and jumping horses. Z. Saeugetierk 54:172–190, 1989
23. Thomason JJ, Biewener AA, Bertram JEA: Surface strain on the equine hoof wall in vivo: implications for the material design and functional morphology of the wall. J Exp Biol 166:145–168, 1992
24. Thomason JJ: Variation in surface strain on the equine hoof wall at the midstep with shoeing, gate, substrate, direction of travel, and hoof shape. Equine Vet J Suppl 26:86–95, 1998
25. Wilson AM, McGuigan MP, Fouracre L, et al: The force and contact stress on the navicular bone during trot locomotion in sound horses and horses with navicular disease. Equine Vet J 33:159–165, 2001
26. Curtis S., Ferguson DF, Luikart CJF, et al: Trimming and shoeing the chronically affected horse. Vet Clin North Am Equine Pract 15:463–480, 1999
27. Merkens HW, Schamhardt HC, Hartman W, et al: Ground reaction force patterns of Dutch Warmblood horses at normal walk. Equine Vet J 18:207–214, 1986
